# Random Search Algorithm for Solving the Nonlinear Fredholm Integral Equations of the Second Kind

**DOI:** 10.1371/journal.pone.0103068

**Published:** 2014-07-29

**Authors:** Zhimin Hong, Zaizai Yan, Jiao Yan

**Affiliations:** Department of Mathematics, Science College of Inner Mongolia University of Technology, Hohhot, P.R. China; University College London, United Kingdom

## Abstract

In this paper, a randomized numerical approach is used to obtain approximate solutions for a class of nonlinear Fredholm integral equations of the second kind. The proposed approach contains two steps: at first, we define a discretized form of the integral equation by quadrature formula methods and solution of this discretized form converges to the exact solution of the integral equation by considering some conditions on the kernel of the integral equation. And then we convert the problem to an optimal control problem by introducing an artificial control function. Following that, in the next step, solution of the discretized form is approximated by a kind of Monte Carlo (MC) random search algorithm. Finally, some examples are given to show the efficiency of the proposed approach.

## Introduction

It is well known that linear and nonlinear integral equations arise in many scientific fields such as the population dynamics, spread of epidemics, thermal control engineering, and semi-conductor devices. Integral equations are usually difficult to solve analytically. Therefore, numeric solutions of integral equations have been a subject of great interest of many researchers. In most cases, the problem of finding numeric solutions for the nonlinear Fredholm integral equations is more difficult than the problem of the linear Fredholm integral equations. The problem of solving numeric solutions for the linear Fredholm integral equations of the second kinds is one of the oldest problems in the applied mathematics literature. Many numerical computational methods including deterministic techniques and Monte Carlo methods are introduced in this area ([1]–[10]). These proposed methods of literatures [1]–[10] have been used to discuss the linear Fredholm integral equations. However, it is difficult to solve the nonlinear kinds with them, 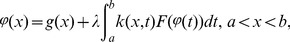
(1)especially analytically, where 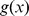 is a known continuous function over the interval 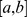, 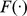 is a known nonlinear function with respect to 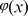 and 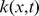 is a kernel function which is known and continuous too, at the same time bounded 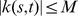 on the square 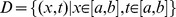 which 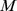 is the upper bound on the square 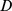. Consequently, our aim here is to find the unknown function 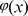 which is the solution of problem (1).

Some deterministic techniques have been applied to obtaining solutions of the nonlinear integral equations during the past several years. Many different numerical and approximate techniques were introduced to obtain the solutions of nonlinear integral equations. Literature [11] applied a different method (DM) to solve the equation (1) by convert the problem to an optimal control problem based on introducing an artificial control function. The results of algorithm [11] depend explicitly on the selection of the starting interval which decreases the speed of convergence substantially. Discrete Adomian decomposition method (DADM) [12] arose when the quadrature rules are used to approximate the definite integrals which can not be computed analytically. Homotopy perturbation method (HPM) introduced by [13] was used to solve the nonlinear integral equations (1). Positive definite function method (PDFM) [14] is based on interpolation by radial basis functions (RBFs) to approximate the solutions of the nonlinear Fredholm integral equations. The accuracy of PDFM relies on the choices of radial basis functions. Triangular functions method (TFM) [15] was utilized as a basis in collocation method to reduce the solutions of nonlinear Fredholm integral equations to the solutions of algebraic equations by using the optimal coefficients. Harmonic wavelet method [16] was employed as basis functions in the collocation method towards approximate solutions of the Fredholm type integral equations. Optimal homotopy asymptotic method (OHAM) was introduced in literature [17] as a reliable and efficient technique for finding the solutions of integral equations. Literature [18] presented a computational method for solving nonlinear Fredholm integral equations of the second kind which is based on the use of Haar wavelets. Iterative method [19] was used to approximate the solutions of the nonlinear Fredholm integral equations (1) by transforming the integral equation into a discretized form. However, each of these methods has its inherent advantages and disadvantages. For some methods, such as iterative method, a good initial value must be given in advance. Otherwise, the obtaining results by the iterative method may diverge or converge to not true solutions of the equations. For some integral equations, the nonlinear function 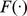 must satisfy convergence conditions of algorithm, otherwise interval of convergence would be local subinterval which is not global interval [19].

In this paper, we intend to present a random numerical scheme for obtaining approximate solutions of the nonlinear Fredholm integral equations (1) by random search algorithm and suppose that the discussed integral equations have one solution at least. At first, we transform the equation into a discretized form by a numerical method of integration, e.g. Simpson's rule.

The present work is motivated by the desire to obtain numerical solutions of the linear Fredholm integral equations of second kind by using Monte Carlo method, which is introduced by Spanier and Gelbard [6], Veach and Guibas [7], Farnoosh and Ebrahimi [8], Zhimin et al. [9] and Doucet et al. [10]. But there have been only a few works that solved the nonlinear Fredholm integral equations such as (1) numerically by Monte Carlo methods.

As a kind of numerical method, Monte Carlo method has a great many merits. For example, it has only a few moments that Monte Carlo method is limited to geometric restrictions, the convergence speed is irrelevant to the dimensions of the problem, the error is easy to determine, the program structure is simple, more flexible and easy to accomplish. Especially, when the solved nonlinear system has a higher dimension, deterministic technique is difficult to determine the solution of system. For numerical problems in a large number of dimensions, Monte Carlo methods are often more efficient than conventional numerical methods.

The paper is divided into five sections. In section 2 we transform the equations (1) into a discretized form by Simpson quadrature. In section 3 Monte Carlo random search algorithm is introduced to solve the numeric solution of nonlinear algebraic system. In section 4 some numerical examples are solved by the proposed method. Section 5 concludes this paper with a brief summary.

## Simpson Quadrature Method

We now approach the subject of numerical integration. The goal is to approximate the part of definite integral in the integral equations (1) over the interval 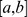 by evaluating integrand 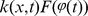 at some sample points.

Consider a uniform partition of the closed interval 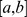 given by 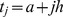 of step length 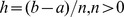 integer, and 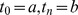, using Simpson quadrature, the integral term 
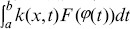
 may be expanded as [20], 
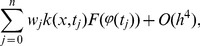
(2)where 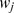 are weights and determined by the quadrature methods.

Further more, the solution of integral equation (1) with quadrature methods is approximated by solving the following system, 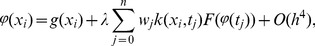
(3)where 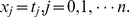


When 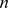 is odd, Simpson's 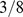 rule is applied to the last three subintervals and then Simpson's 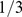 rule is applied to the remained subintervals.

Denoting the notation 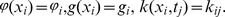



If the truncation error of equation (3) is neglected, the nonlinear algebraic system can be obtained, as follows, 
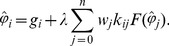
(4)


If let 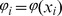 be an exact solution of equation (1) at point 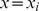, then 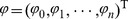 is also a solution of equation (3). Denoting 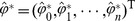 as a solution vector of nonlinear system (4).

The conditions that 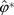 approaches to exact solution 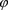 will be given by the following corollary [19].

**Corollary 1** *Suppose the following conditions*

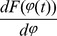
 *exists for every* 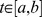,

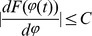
, 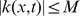, 
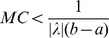
,

*hold, then*

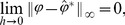
(5)where 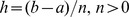.

### Proof

Please refer to literature [19].

For nonlinear algebraic system (4), Monte Carlo method will be used to obtain the approximate numeric solutions of (4) in the next section. The program structure of this method is simple, flexible and easy to accomplish. For problems whose required precision is relatively high, Monte Carlo method is a very good choice to achieve a good initial value.

## Monte Carlo Random Search Algorithm

With regard to the nonlinear algebraic system (4), we introduce notation 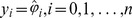 that simplifies our work and system (4) can be rewritten as 
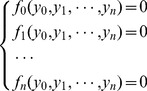
(6)where 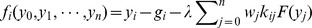.

Denoting 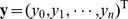, by equation (6), we establish objective function: 
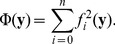
(7)


Obviously, if 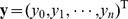 is the solution of equation (6), then 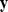 also makes the objective function 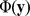 equal zero. Hence, the unknown solution 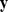 of nonlinear equation (6) can be determined in such a way that the objective function 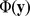 is minimized. Namely, suppose that there is a vector 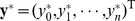 such that 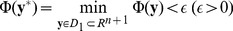
(8)holds, then 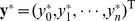 is not only an approximation solution of nonlinear equation (6), but also an approximation solution of nonlinear integral equation (1) at point 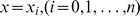 when the kernel function 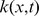 and nonlinear function 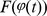 satisfy the conditions of Corollary 1.

In this paper, numerical methods based on random sampling, especially Monte Carlo methods, can be applied to this optimization problem.

For solving problem (6), we consider Monte Carlo random search algorithm in Table 1.

**Table 1:** Algorithm 1.

Step	Operation
1	Choose 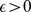 , the maximum number 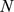 of random search and step size 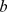 of random search. Select an initial solution 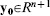 where 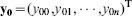 and 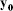 can be randomly selected by some probability distributions.
2	Set 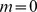 .
3	Compute 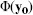 .
4	If 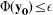 , go to step 10.
5	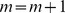 , if 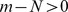 then set 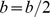 and 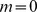 .
6	Generate 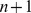 -dimensional random variable 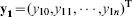 from a normal distribution, that is 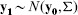 , 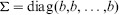 .
7	Compute 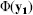 , if 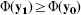 , go to step 5.
8	Set 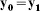 and 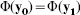 .
9	Go to step 4.
10	If the stopping criterion is met, then stop

## Numerical Examples

In this section, we are going to demonstrate some numerical results for solving 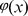 in the nonlinear problem (1). Therefore the following examples are considered and the numerical solutions are obtained based on Monte Carlo random search algorithm. In order to measure the efficiency of the proposed MC random search algorithm, the symbolic solution is also used to solve the following examples.

**Remark:** In Table 2 and Table 3, results of error 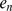 of the symbolic solution here is referring to the truncation error of equation (3).

**Table 2:** Errors of numerical results 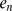 of Example 1 and Example 2.

	Example 1: 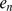		Example 2: 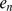	
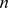	MC	Symbolic solution	MC	Symbolic solution
3	0.000317436	0.000312506	0.000779989	0.000780082
5	2.37532E-05	2.33971E-05	8.86961E-05	8.85708E-05
7	3.59133E-06	3.53349E-06	2.08152E-05	2.08137E-05
9	8.69071E-07	7.67474E-07	7.06189E-06	7.06065E-06
13	1.14246E-07	1.07254E-07	1.46446E-06	1.46353E-06

**Table 3:** Errors 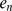 of simulated results for methods PDFM [14], DM [11] and iterative method [19] in Example 1 and Example 2.

	Example 1: 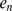		Example 2: 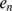		
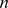	MC	[14]RBF( 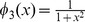 )	MC	DM [11]	[19]( 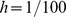 )
5	2.4E-05	1.7E-03 ( 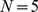 )	8.7E-05		
10	8.7E-07	2.1E-05 ( 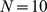 )	7.1E-06	2.0E-02	7.0E-03
15	7.2E-10	1.5E-08 ( 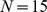 )	7.6E-07		

Let 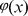 be exact solution of the nonlinear Fredholm integral equation (1), 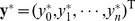 (where 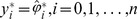) is an approximation solution obtained by using the given Table 1 with a known 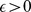 and partition step size 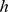 and 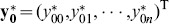 is an initial value by random sampling. In all examples (except Example 3), we choose the initial value 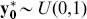. In Example 3, we set up the initial value 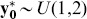. By reference [14], we define the maximum error to measure the efficiency of MC method for different values 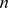, which is given as 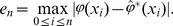
(9)


### Example 1

Consider the following nonlinear Fredholm integral equation [14]

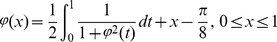
which has the exact solution 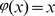.

Errors of the numerical results for different values 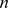 are given in Table 2. Table 3 lists comparison of results with PDFM [14].

In this example, for reference [14], as radial basis functions (RBF) take different forms, the accuracy based on RBF is different. For instance, as radial basis functions 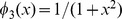, the maximum error is 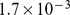 with 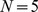. But for our proposed method in this paper, from Table 2, the maximum error is 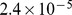 with the same value of 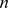.

### Example 2

Consider the following nonlinear integral equation [11], [19]

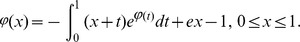



For which the exact solution is 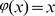.

One may find in Table 2 the comparison of exact and obtained approximate solutions for different values 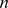. Comparison of results with DM [11] and iterative method [19] is displayed in Table 3.

Comparison of the results of this example with those obtained in [11] and [19] shows the efficiency of the proposed MC algorithm more accurate.

### Example 3 [16, 18]



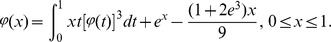



For which the exact solution is 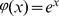. In this example, for symbolic solution, if we set up the initial value 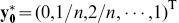, symbolic solution is failure. But for Monte Carlo random search algorithm, the order of maximum error 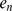 is still 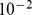.

Table 4 shows errors of the numerical results for Example 3 with different values 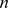. Table 5 shows that the proposed Monte Carlo method solves equation (1) more accurately than methods harmonic wavelets [16] and Haar wavelets [18].

**Table 4:** Errors of numerical results 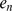 of Example 3 and Example 4.

	Example 3: 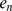		Example 4: 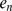	
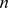	MC	Symbolic solution	MC	Symbolic solution
3	0.031940214	0.031942343	0.009557852	0.009557697
5	0.004775551	0.004748677	0.00391024	0.003910249
7	0.001225184	0.00122517	0.000631981	0.000631981
9	0.000432635	0.000432178	9.44288E-05	9.44288E-05
13	9.16911E-05	9.16792E-05	4.48755E-06	4.48744E-06

**Table 5:** Errors 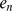 of simulated results in Example 3 based on harmonic wavelet [16] and Haar wavelets [18] methods.

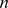	MC	harmonic wavelets: 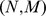	Haar wavelets: 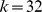
8	1E-04	9E-02 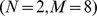	
16	2E-05	8E-03 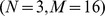	2E-02
20	9E-06	8E-07 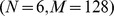	

### Example 4 [13, 15, 17]



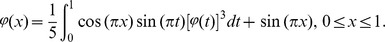



In this example, the analytical solution of this equation is 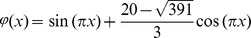 on interval 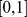.

Table 4 shows errors of the numerical results for Example 4 with different values 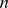. Table 6 describes comparison of maximum errors 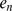 with HPM [13], TFM [15] and OHAM [17]. From comparison of the results of Example 4, we see that MC method provides a better numerical solution for the nonlinear Freholm integral equation of second kind and is more preciser than deterministic methods HPM and TFM. The proposed MC method and OHAM [17] can have the same order of error, is 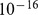 as 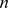 is large.

**Table 6:** Errors 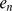 of simulated results for methods HPM [13], TFM [15] and OHAM [17] in Example 4.

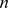	MC	HPM [13]( 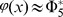 )	TFM [15]( 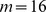 )	OHAM [17]
4	8.3E-03			
16	9.2E-16	1.2E-06	2.0E-08	2.5E-16
20	2.8E-16			

### Example 5 [19]



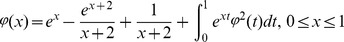
which has the exact solution 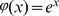.

**Figure 1:**
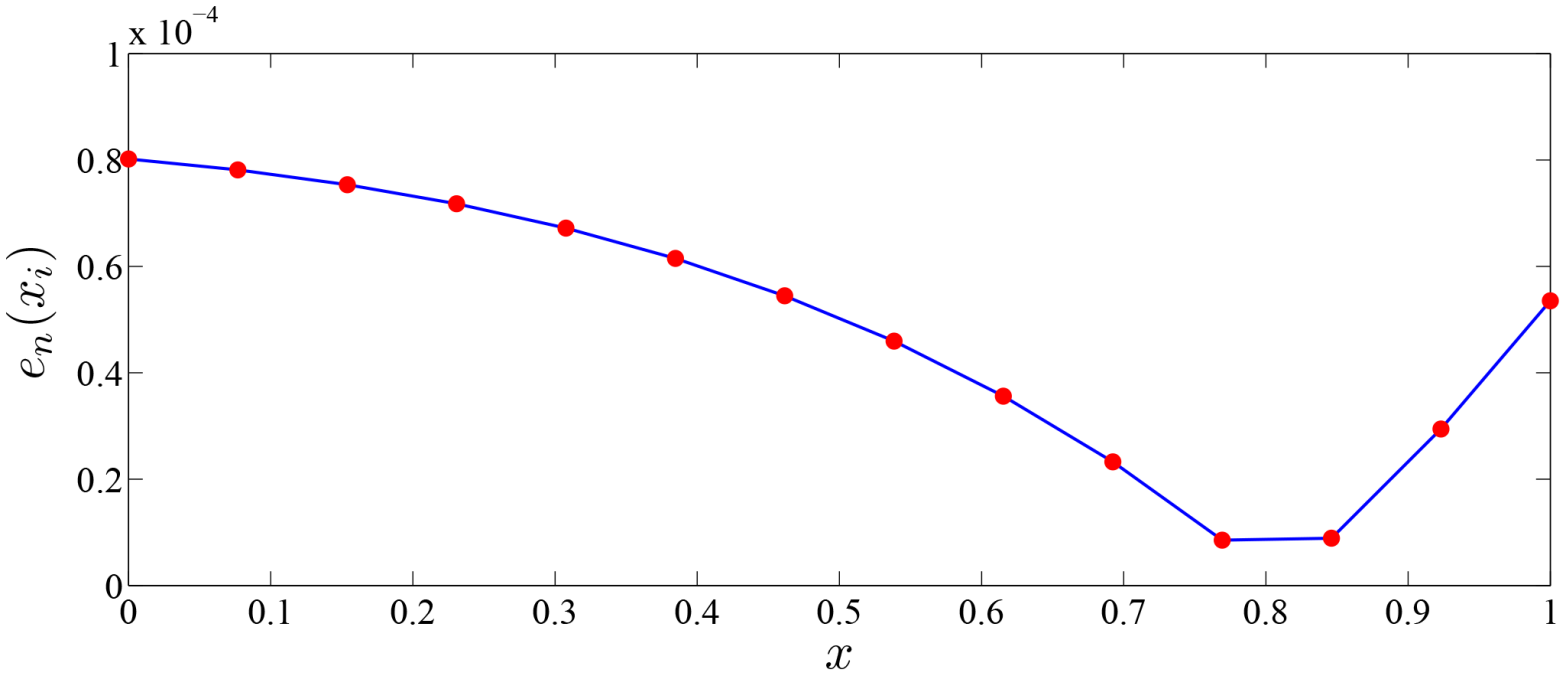
Errors of MC random search with 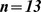 and variant value 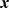 in Example 5.

The error function 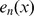 in Figure 1 shows the precision of the approximate solution in example 5. The result shows that the interval of convergence of the proposed MC method is global interval 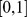 and the order of error is 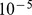. But for reference [19], the interval of convergence of the deterministic method is local subinterval 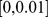 and the order of error is 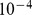.

### Example 6

Consider the following nonlinear integral equation [21]

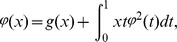
where 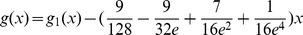,



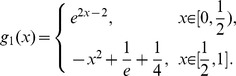



The exact solution is 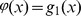. Table 7 illustrates the numerical results of Example 6 using methods of [21] and the proposed MC.

**Table 7:** Errors 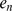 of simulated results for Legendre wavelets method [21] in Example 5.

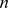	MC	Legendre wavelets method	Legendre wavelets method
4	7.6E-04		
16	4.6E-06	1.8E-03( 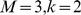 )	1.3E-04( 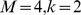 )
20	1.9E-06		

### Example 7

As the final example we consider a physical problem which is of great interest in magnetohydrodynamics [22].

The proposed MC method is used to solve an integral equation reformulation of the nonlinear two-point boundary value problem 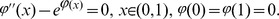



This problem has the unique solution 
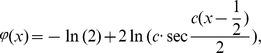
where 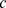 is a unique solution of 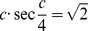 and this nonlinear two-point boundary value problem may be reformulated as the integral equation



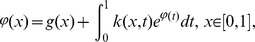
where the free term 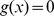, kernel function 
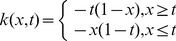
 is the Green's function for the homogeneous problem



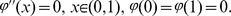



The errors results using the method of [23] together with the results obtained for errors 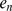 by the proposed MC method are tabulated in Table 8.

**Table 8:** Error results for Example 6.

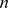	MC	Method of [23]
5	5.8E-03	5.2E-04
9	1.9E-03	1.3E-04
17	5.5E-04	3.2E-05

**Figure 2:**
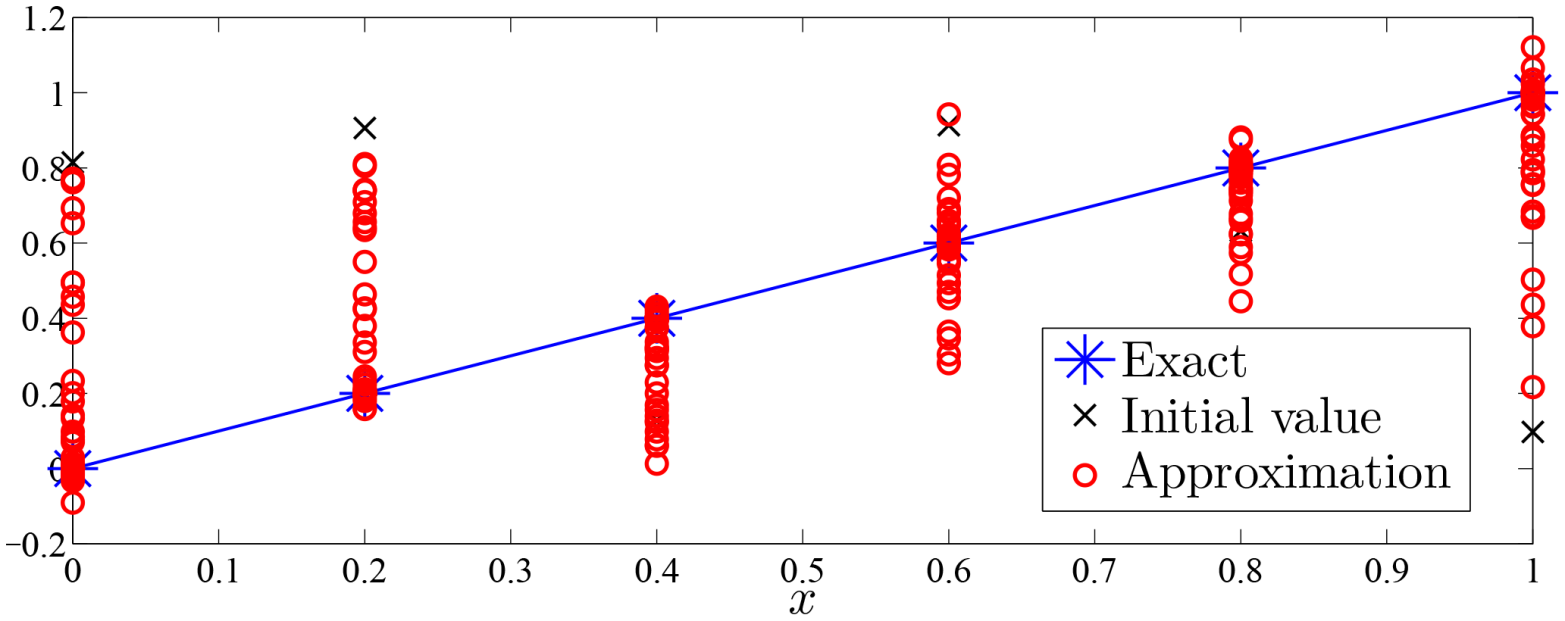
Approximation process of MC random search with 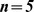 in Example 1 for solving 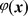.

**Figure 3:**
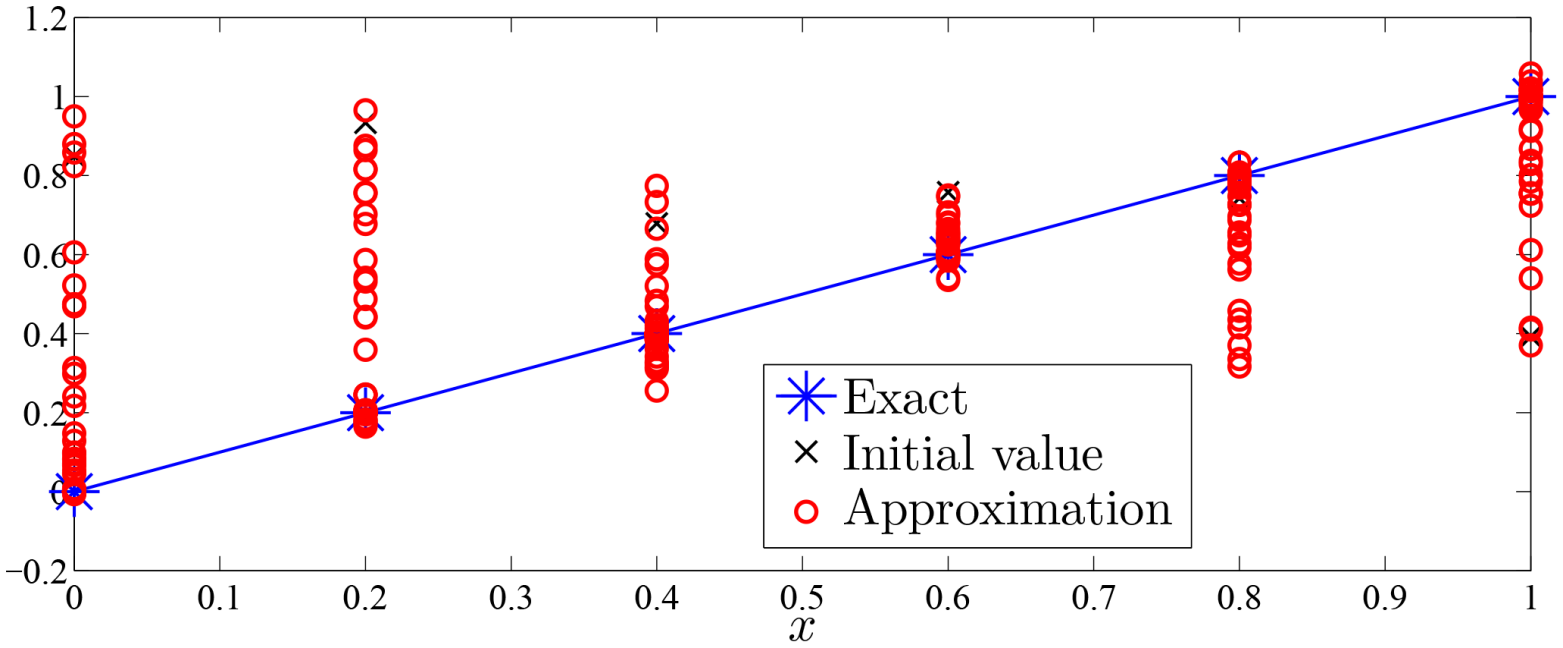
Approximation process of MC random search with 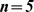 in Example 2 for solving 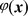.

**Figure 4:**
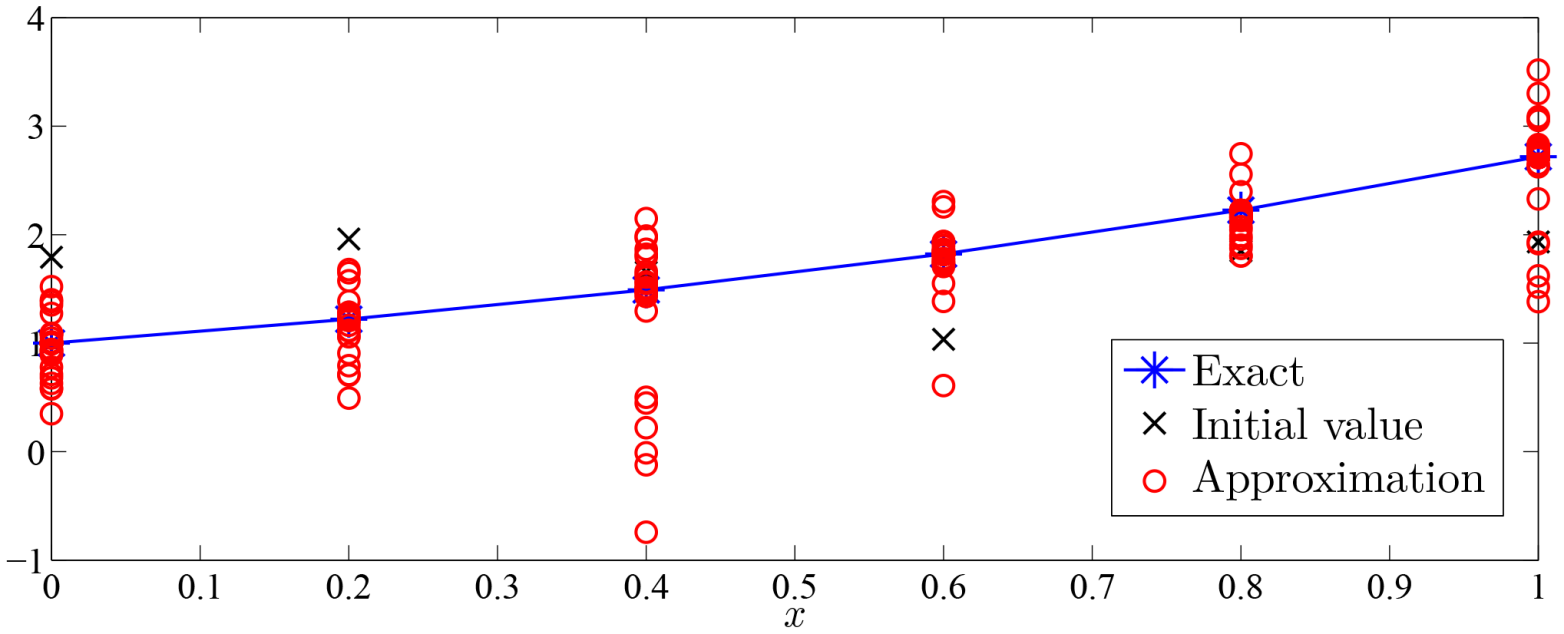
Approximation process of MC random search with 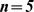 in Example 3 for solving 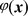.

**Figure 5:**
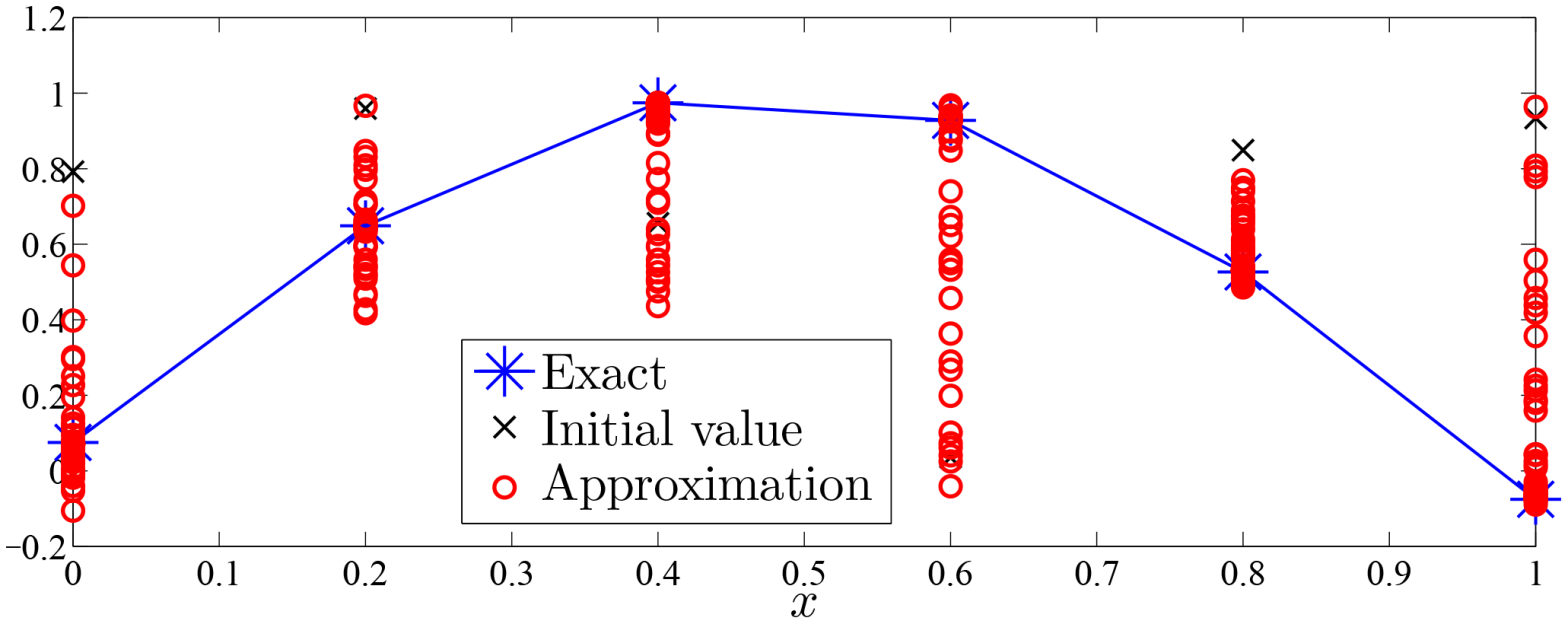
Approximation process of MC random search with 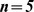 in Example 4 for solving 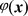.

**Figure 6:**
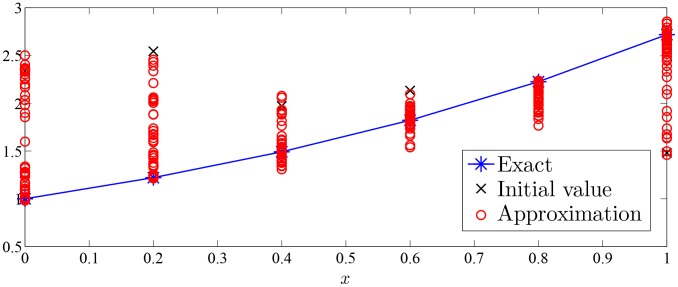
Approximation process of MC random search with 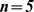 in Example 5 for solving 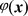.

**Figure 7:**
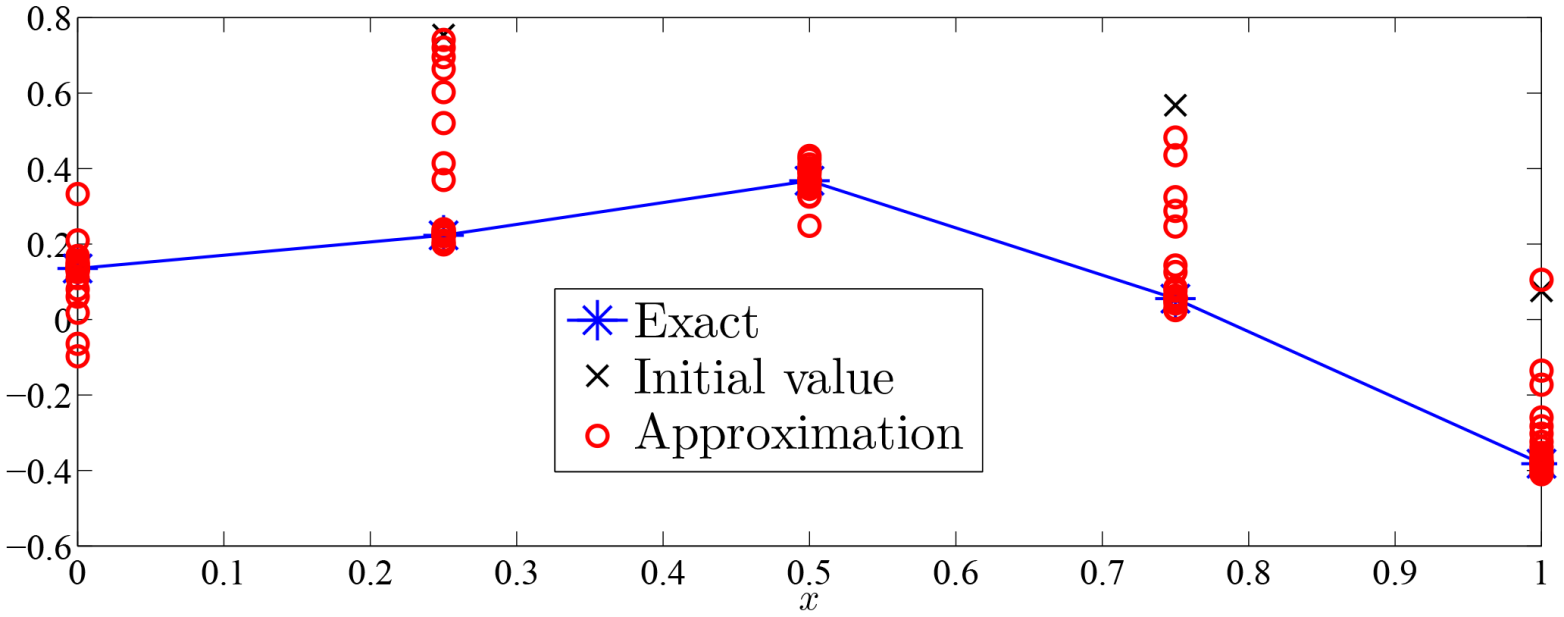
Approximation process of MC random search with 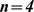 in Example 6 for solving 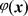.

**Figure 8:**
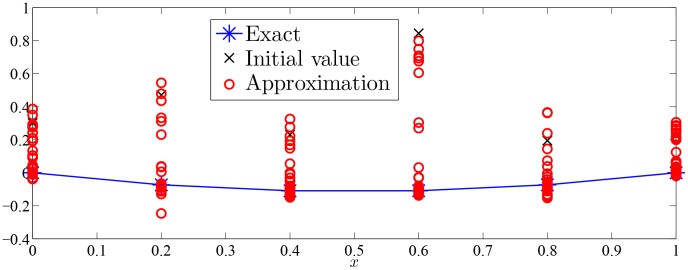
Approximation process of MC random search with 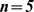 in Example 7 for solving 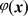.

Approximation process of Monte Carlo random search algorithm of Examples 1–7 were drawn with given value of 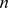 in Figures 2–8.

As was seen in Figures 2–8, numerical solution of equation (1) based on the proposed Monte Carlo random search algorithm is convergent in probability to the analysis solution and this proposed MC method is stable.

## Conclusion

In this paper, a numerical method based on quadrature methods and Monte Carlo random search algorithm has been proposed to approximate the solutions of the nonlinear Fredholm integral equations. With this method, the problem of solving integral equation is reduced to a problem of solving a nonlinear system of algebraic equation. Illustrative examples are given to demonstrate the validity and accuracy of the proposed MC method. In Tables 1, 3, solutions of Examples 1–4 are presented for different values of 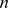 to show that the maximum errors 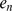 based on the proposed MC method and the direct solving method of equation (4) have the same order. The results obtained by MC random search algorithm are compared with solutions obtained by DM, DADM, HPM, PDFM, TFM, OHAM, harmonic wavelets, Haar wavelets, Legendre wavelets, iterative method and collocation-type method. Tables 2, 4, 5, 6, 7 and Figure 1 show that the proposed MC method is reliable and efficient for finding the solutions of integral equations in Examples 1–7. Figures 2–8 shows that the propose MC method is convergent and stable. For concrete problems that the region of root of integral equation (1) is unknown, the proposed Monte Carlo random search algorithm is a good and reliable choice to obtain an initial value and achieve a high precision when we deal with the problems. By the way the program structure of this method is simple and easy to accomplish.
